# Pott's puffy tumor: A case report

**DOI:** 10.1002/ccr3.7815

**Published:** 2023-10-17

**Authors:** Gholamreza Vadiee, Mohamad Beshali, Soodeh Jahangiri, Zahra Eghlidos, Zahra Rahimian, Farhad Mirzaei

**Affiliations:** ^1^ Research Center for Neuromodulation and Pain Shiraz Iran; ^2^ Department of Neurosurgery, Faculty of Medicine Tabriz University of Medical Sciences Tabriz Iran; ^3^ Endocrine Research Center, Institute of Endocrinology and Metabolism Iran University of Medical Sciences Tehran Iran; ^4^ Student Research Committee Shiraz University of Medical Sciences Shiraz Iran

**Keywords:** forehead swelling, osteomyelitis, Pott's edematous tumor, Pott's puffy tumor

## Abstract

**Key Clinical Message:**

Pott's puffy tumor is a rare condition primarily occurring in the younger population. This report highlights the clinical suspicion and diagnosis of Pott's puffy tumor in those presenting with favorable presentations, especially adolescents.

**Abstract:**

Pott's puffy tumor (PPT) is characterized as frontal bone subperiosteal abscess and osteomyelitis, a rare condition primarily occurring in adolescents following frontal sinusitis or head trauma. We present a case of atypical PPT in a 12‐year‐girl following an insect bite. The patient presented with painful forehead swelling for 4 weeks without any history of head trauma or signs of sinusitis. She had a history of a purulent pimple 2 months before presentation, following an insect bite. The primary diagnosis of PPT was made based on clinical and imaging findings. The patient was treated surgically and medically with intravenous antibiotics and had a satisfactory recovery upon the 6‐month follow‐up visit. This case highlights the differential diagnosis and thorough evaluation for PPT in a child with acute headache and forehead swelling, even without sinusitis symptoms.

## Graphical abstract

CT scan and MRI of the patient. Extra‐axial mass with compressive effects is evident in the frontal lobe.

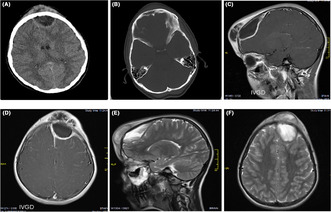

## 1 INTRODUCTION

Pott's puffy tumor (PPT) is the frontal bone subperiosteal abscess with frontal bone osteomyelitis. Affected individuals present with swelling, inflammation, and puffiness of the forehead to the frontal area; the common underlying causes are forehead trauma and frontal sinusitis.^1^ It is a rare condition, primarily occurring in adolescents. An early diagnosis and management are crucial to prevent further complications including but not limited to subdural/epidural empyema, meningitis, encephalitis, and frontal lobe abscess.^2^

This report describes an atypical presentation of PPT in an adolescent, with painful forehead swelling after an insect bite, without sinusitis symptoms or any history of trauma.

## 2 CASE REPORT

A 12‐year‐old village‐living girl was referred to our center with a complaint of progressive painful swelling in the forehead area for 1 month. The patient did not mention any history of head trauma or infection. According to her parents, she only had a history of a purulent pimple on her forehead 2 months prior to the visit and after an insect bite. This pimple healed on its own and then the forehead swelling appeared. In the clinical examination, vital signs were normal without signs of distress. A lump‐shaped swelling measuring 9 × 5 cm was observed with a relatively shiny appearance, and it was hard and painful to the touch. There were no focal neurological findings. The paraclinical examinations showed, an erythrocyte sedimentation rate of 25 mm/h (normal level: 0–22 mm/h) and a white blood cell count of 9800 × 109/L (normal level: 4.5–11.0 × 109/L).

**FIGURE 1:**
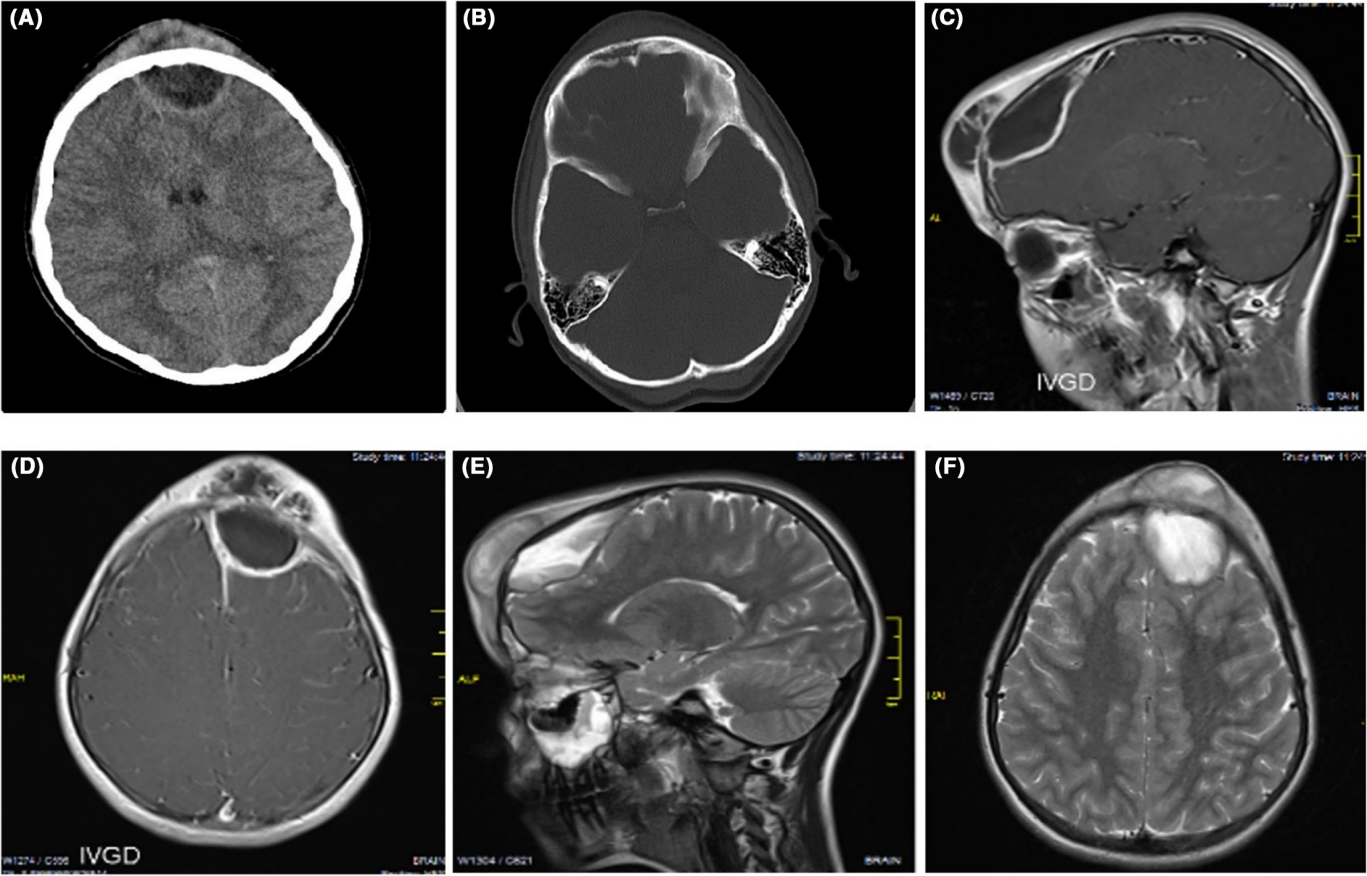
CT scan and MRI imaging of the patient. (A) Axial brain CT scan showing swelling of the overlying scalp and a hypodense frontal lobe abscess with a hyperdense rim. (B) Axial brain CT scan indicating erosion of the left frontal bone and opacification of the frontal sinus. (C and D) Contrast enhanced T1‐weighted sagittal and axial brain MRI sections, respectively, showing Pott's puffy tumor and intracranial extradural abscesses. (E and F) T2‐weighted sagittal and axial brain MRI sections showing a subperiosteal abscess in the forehead and an epidural abscess.

In a non‐contrast computed tomography (CT) scan, a hypodense mass was seen in the frontal and subgaleal areas. Another hypodense mass was present in the intracranial frontal region with a distinct border and destruction of the opposite bone tissue. For additional examination of the involved intracranial tissue, magnetic resonance imaging (MRI) with and without contrast material was performed; then the lesion was identified as a heterogeneous extra‐axial mass with a compressive effect and a well‐defined ring enhancement in the frontal lobe (Figure 1).

According to the clinical examinations and paraclinical and imaging findings, the primary diagnosis of PPT was made. A bi‐frontal craniotomy was performed with a bi‐coronal skin incision. Immediately after opening the skin, much purulent fluid was removed, and a sample was collected for culture and staining. A granulation tissue was formed under the galea, having an apparent adhesion to the underlying cranial bone which was separated. Puss was coming out of small holes formed in the frontal bone. After creating the first burr hole, large amounts of dark creamy purulent fluid were released, and samples were sent for culture. After separating the bone flap, we thoroughly treated the area below the bone with 2 L of normal saline containing gentamicin. We ensured that the dura was intact and removed the deformed parts of the internal surface of the skull bone with a high‐speed bur as far as possible. Then the bone flap was placed back, the scalp was sutured in layers, and a drain was inserted. The pathobiology laboratory of the center later reported that the sample had polybacterial growth including streptococci, anaerobes, and staphylococci. We decided on the empirical antibiotic regimen after consultation with the infectious disease specialists and the familiarity of treatment resistance in our center; intravenous ceftazidime 2 grams every 12 h, vancomycin 500 mg every 6 h, and metronidazole 400 mg every 8 h for 6 weeks was started. The patient did not have any complications or recurrence in the follow‐up 6 months after the surgery.

## 3 DISCUSSION

Since 1768, several cases of PPT have been reported, although it is uncommon to see severe cases after the advent of antibiotics in the treatment of infectious diseases. This disease covers a wide age range, but in children, it may be due to gradual developmental changes in frontal sinus anatomy concerning pneumatization. Also, structural abnormalities during the pneumatization process, such as over‐pneumatized ethmoid bullae, extensively pneumatized middle turbinate, or enlargement of agger nasi cells, by creating anatomical disorders caused by blockage or movement disorder of the cilia, cause inflammation of the mucosa and create a suitable environment for the growth of anaerobic bacteria.^3^, ^4^, ^5^

Frontal sinus infection is the most common cause; however, forehead trauma can also be one of its common causes.^6^, ^7^ Other rarely reported causes are burns in the cranial or frontal region, tooth infection, cocaine use, injuries caused by sports competitions such as wrestling, and insect bites.^8^, ^9^ In some studies, factors such as diabetes, aplastic anemia, chronic kidney disease, and other immunosuppressive factors have been mentioned as risk factors.^4^

In most cases, culture results are reported as polymicrobial. Non‐enterococci streptococci (47%), anaerobic oral bacterial (28%), and staphylococci (22%) organisms are common, while Fusobacterium, H. influenza, Enterococcus, Pseudomonas, Escherichia Coli, Pasteurella multocida, Proteus, and Bacteroides has been reported more rarely.^10^, ^11^, ^12^

The pathophysiology of frontal bone osteomyelitis in PPT disease can be caused by direct involvement of the infection or by hematogenous spread, which takes place through the connection of the frontal sinus with the dural venous networks and connection to the diploic veins.^1^, ^4^, ^13^

The most common symptoms of the disease are forehead swelling, headache, congestion, and purulent or non‐purulent discharge from the nose in the form of rhinorrhea.^11^, ^14^, ^15^ Fever and erythematous forehead swelling are considered pathognomonic of PPT but fever can be absent in some cases.^13^ Other rare symptoms of this disease include focal neurological signs, nausea and vomiting, skin fistula, epidural empyema, meningitis, encephalitis, cranial nerve involvement, photophobia, seizures, and changes in the level of consciousness. In case of acute neurological symptoms, emergency treatment should be done,^1^, ^11^, ^14^

The diagnosis is based on clinical suspicion, and the next steps are confirmed by paraclinical tests and imaging. Although a contrast‐enhanced CT scan of the head seems to be sufficient, contrast‐enhanced MRI can reveal more details of intracranial soft tissue involvement and the extent of infection or dural involvement, which may cause dural sinus thrombosis.^1^, ^4^, ^11^, ^12^, ^13^, ^14^, ^16^, ^17^, ^18^ The dura and arachnoid membrane act as barriers against the penetration of infection into the underlying tissue, nevertheless subdural empyema and cerebritis have been reported as complications of PPT.^19^, ^20^ Therefore, timely diagnosis and treatment prevent complications and risks in patients. The best treatment approach is the combination of surgery to visualize the abscess, remove the destroyed tissue, and restore the drainage of the sinuses, sinusectomy, and systematic antibiotic treatment.^1^, ^4^, ^12^, ^14^, ^16^, ^21^, ^22^

As soon as the diagnosis is suspected, an empirical broad‐spectrum antibiotic regimen with good penetration into intracranial tissue should be started. Penicillin, third‐generation cephalosporin, vancomycin, and metronidazole are some examples. More targeted changes should be applied after culture affirmation. Antibiotic treatment should be continued intravenously for a period of 4–8 weeks.^23^, ^24^, ^25^

PPT after an insect bite was previously reported by Raja and colleagues^8^ in an adult patient; a 49‐year‐old male who presented with right upper eyelid swelling following an insect bite. This persisted into right forehead swelling in the follow‐up examination. Upon CT examination soft tissue swelling and edema, bony erosion, and destruction of frontal bone were observed.

Overall, the design of the study (i.e., case report) limits us from generalizing our findings to other settings; however, the rising number of case studies reporting PPT, highlights the importance of this diagnosis among patients with suggestive presentation.

## 4 CONCLUSION

In conclusion, although PPT is rarely reported, it may occur so the clinical suspicion and timely diagnosis of PPT especially in children with favorable symptoms is crucial to prevent its dangerous complications.

## AUTHOR CONTRIBUTIONS

**Gholamreza Vadiee:** Data curation; formal analysis; project administration. **Mohamad Beshali:** Data curation; formal analysis; project administration. **Soodeh Jahangiri:** Writing – original draft; writing – review and editing. **Zahra Eghlidos:** Writing – original draft; writing – review and editing. **Zahra Rahimian:** Writing – original draft. **Farhad Mirzaei:** Conceptualization; supervision.

## FUNDING INFORMATION

None.

## CONFLICT OF INTEREST STATEMENT

The authors have no conflict of interest to declare.

## ETHICS APPROVAL AND CONSENT TO PARTICIPATE

According to the ethics committee of the Tabriz University of Medical Sciences, ethical approval was not required for the study. The patient's parents gave informed consent to participate in this study.

## CONSENT FOR PUBLICATION

Informed consent was obtained from the patient and parents for the publication of anonymous imaging or data.

## Data Availability

The datasets used and/or analyzed during the current study are available from the corresponding author upon reasonable request.
